# Infection control, prophylactic antibiotics, and testing for SARS-CoV-2 and PPE on German intensive care units: results from a national mixed methods survey

**DOI:** 10.3205/dgkh000392

**Published:** 2021-06-14

**Authors:** Steffen Dickel, Clemens Grimm, Maria Popp, Claudia Struwe, Alexandra Sachkova, Martin Golinski, Christian Seeber, Falk Fichtner, Daniel Heise, Peter Kranke, Winfried Meissner, Sven Laudi, Sebastian Voigt-Radloff, Joerg J. Meerpohl, Jonas Jabs, Nico T. Mutters, Onnen Moerer, German CEOsys Study Group

**Affiliations:** 1 Department of Anesthesiology and Intensive Care Medicine, University Medical Center Göttingen, Göttingen, Germany; 2 Department of Anaesthesiology, Intensive Care, Emergency and Pain Medicine, University Hospital of Würzburg, Würzburg, Germany; 3 Department of Anesthesiology and Intensive Care Medicine, University Medical Center Leipzig, Leipzig, Germany; 4 Department of Anesthesiology and Intensive Care Medicine, University Medical Center Jena, Jena, Germany; 5 Institute for Evidence in Medicine, Medical Center & Faculty of Medicine, University of Freiburg, Freiburg, Germany; 6 Cochrane Germany, Cochrane Germany Foundation, Freiburg, Germany; 7 University Hospital Bonn, Institute for Hygiene and Public Health, Bonn, Germany

**Keywords:** COVID-19, PPE, prophylactic antibiotics, variants of concern, cohort isolation

## Abstract

**Aim:** Recommendations on hygiene measures, personal protective equipment (PPE), isolation, and antibiotic prophylaxis were developed during the coronavirus 2019 disease (COVID-19) pandemic and have been revised several times to date. Some of the underlying literature indicates a large evidence gap. We suspect that this leads to a large variance of measures on German intensive care units (ICU).

**Methods:** A mixed methods online survey among intensive-care specialists in Germany caring for COVID-19 patients was conducted in December 2020.

**Results:** We received responses from 205 German ICUs that had treated COVID-19 patients to date. There was wide variation in the use of PPE. Polymerase Chain reaction (PCR) testing for *severe acute respiratory syndrome coronavirus type 2* (SARS-CoV-2) was used by 94.8% of the units, with an average waiting time of 12 hours for the result. 18.7% of the respondents prescribed antibiotic prophylaxis in COVID-19 patients.

**Conclusion:** We found a high variance in essential care strategies for COVID-19 patients on German intensive care units. This included differences in infection prophylaxis, personal protective equipment, and the indication of prophylactic antibiotic therapy. Based on our results, we recommend further studies to quantify and improve guideline adherence.

## Zusammenfassung

**Zielsetzung:** Empfehlungen zu Hygienemaßnahmen, persönlicher Schutzausrüstung (PSA), Isolierung und Antibiotikaprophylaxe wurden während der COVID-19 Pandemie entwickelt und bis heute mehrfach überarbeitet. Ein Teil der zugrundeliegenden Literatur weist eine große Evidenzlücke auf. Wir vermuten, dass dies zu einer großen Varianz der Maßnahmen auf deutschen Intensivstationen führt.

**Methoden:** Mixed-methods-Onlineumfrage unter leitenden Intensivmedizinern in Deutschland, die COVID-19-Patienten betreuen, durchgeführt im Dezember 2020.

**Ergebnisse:** Wir erhielten Antworten von 205 deutschen Intensivstationen, die bis zu diesem Zeitpunkt COVID-19-Patienten behandelt hatten. Es gab eine große Variation in der Verwendung von PSA. Die Polymerase-Kettenreaktion (PCR) zum Nachweis von SARS-CoV-2 wurde von 94,8% der Stationen verwendet, wobei die durchschnittliche Wartezeit auf das Ergebnis 12 Stunden betrug. 18,7% der Befragten begannen bei COVID-19-Patienten mit einer Antibiotikaprophylaxe.

**Schlussfolgerung:** Wir konnten eine hohe Versorgungsvarianz in Hinblick auf essentielle Versorgungsstrategien von COVID-19 Patienten auf deutschen Intensivstationen feststellen. Hierzu zählten Unterschiede im Bereich der Infektionsprophylaxe, persönlicher Schutzausrüstung und der Indikationsstellung einer prophylaktischen Antibiotikatherapie. Auf Basis unserer Ergebnisse empfehlen wir weitere Untersuchungen zur Quantifizierung und Verbesserung der Leitlinienadhärenz.

## Introduction

The SARS-CoV-2 pandemic has brought unprecedented challenges to medical care. This is particularly evident in the complex treatment of intensive care patients. In addition, due to the high number of patients during the pandemic disease waves in recent months, increased organizational complexity and limited capacity have put the German hospitals and health care workers (HCW) under pressure [1], [2], [3]. Various experts and organizations, including the Robert Koch Institute (RKI), recommend accommodating patients with infectious diseases in a single room with an airlock and a private bathroom [4], [5]. Implementing these requirements appears difficult due to the high number of cases and the insufficient number of isolation wards and single rooms in German hospitals [6]. Another major challenge lies in the timely identification of SARS-CoV-2 positive, and thus potentially infectious, patients. Polymerase chain reaction testing is considered the gold standard in this regard, but is associated with a non-negligible waiting period until results are available [7].

Especially at the beginning of the pandemic, there was a pronounced lack of personal protective equipment, both internationally and nationally [8], [9], [10], [11]. Numerous recommendations for protection against infection among medical personnel have since been published [4], [12], [13]. The de-isolation of patients also represents an important aspect in the care for patients with COVID-19 [14], [15]. Although SARS-CoV-2 RNA is detectable for up to 12 weeks after recovery [16], [17], infectivity is by no means self-evident. For patients with mild to moderate courses, no replicable virus was detected after 10 days [18], [19], [20], [21]. Furthermore, no transmission beyond 6 days after disease onset was documented during contact tracing [22]. In contrast, replicable virus has been isolated in severe cases for up to 20 days [23] and even beyond in severely immunosuppressed patients [24], [25], [26], [27], [28]. As a consequence, depending on the course of the disease and history of the patient, an individual strategy must be applied.

Based on a national survey, we report the current practice regarding isolation, testing and de-isolation of patients with SARS-CoV-2 infection in German ICUs. We highlight different aspects regarding current research results, e.g., the so-called VoC (variants of concern) and their increasing global and national spread. 

## Methods

The data presented stems from a mixed method online survey conducted between December 3 and 31, 2020 during the 2^nd^ wave of the COVID-19 pandemic in Germany. The survey was prepared within the framework and with the expertise of CEOsys, a German research network on COVID-19 [29]. Data related to general COVID-19-related intensive care and staffing are published elsewhere. The invitation to the underlying survey was sent by email to all members of the *German Interdisciplinary Association for Intensive Care Medicine* (DIVI) email distribution list and was addressed to leading intensive-care specialists. A total of 205 ICUs involved in the treatment of COVID-19 patients participated. 

### Survey format

The format of the entire survey included 36 to 44 multiple choice, multiple select, and free text questions, depending on the answers given (adaptive questioning), with 3 to 5 questions per page. The survey focused primarily on current practice in the treatment of COVID-19 patients in German intensive care units. The detailed questions, including results on personal protective equipment, testing, and hygiene, can be found in Table 1 (Tab. 1). 

### Data safety and ethics

Participation in the survey was completely voluntary and anonymous. For this reason, non-participation did not lead to disadvantages. No rewards or money were offered. The participants had the option of providing their email address in order to cooperate in later research projects. The email address cannot be linked to the data collected afterwards

## Results

**Figure 1:**
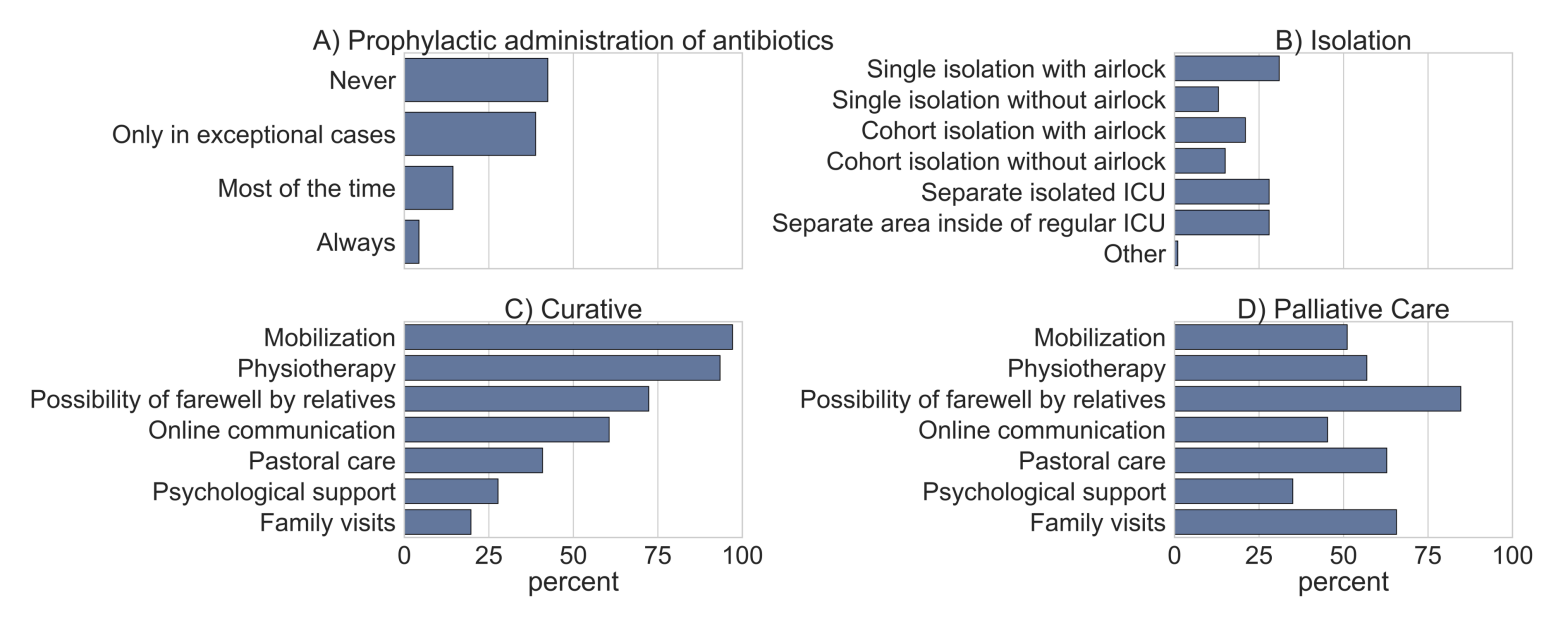
Results of selected survey questions. (A) Management of prophylactic antibiotic administration. (B) Isolation measures in COVID-19 patients. (C) Supportive measures offered to critically ill COVID-19 patients in a curative setting in an unchanged extent. (D) Supportive measures offered to critically ill COVID-19 patients in a palliative setting in an unchanged extent.

**Figure 2:**
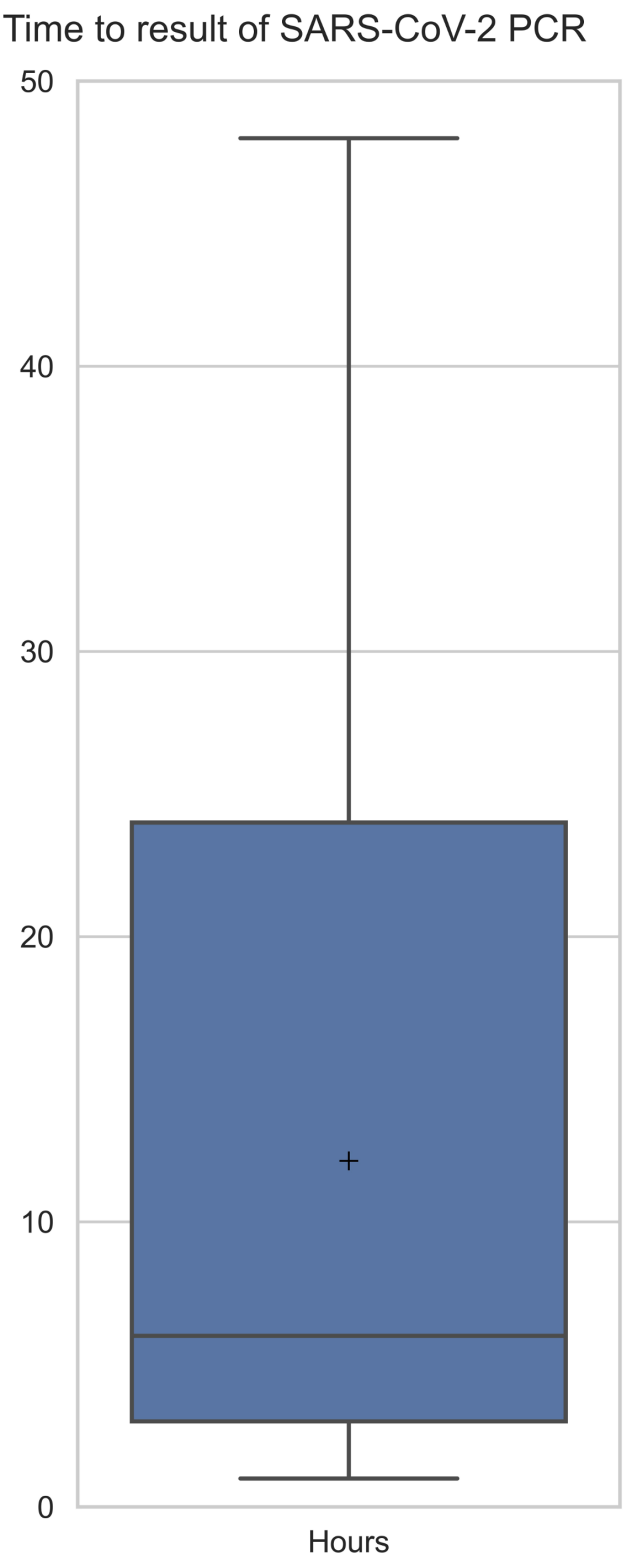
Average time an ICU has to wait for the COVID-19 PCR test result.

We received responses from 244/1,340 (~18%) ICUs registered in the national DIVI registry. Of these 244 ICUs, 205 units treated COVID-19 patients, whose data were included in this study. 66.3% of all participants answered all questions. This required an average of 9:07 minutes (mean value). 135 ICUs responded to questions about infection control, PPE, prophylactic usage of antibiotics and de-isolation practices. This resulted in a completion rate of 65.8%. The detailed results of the study are shown in Table 1 (Tab. 1), Figure 1 (Fig. 1), and Figure 2 (Fig. 2).

### Isolation measures

20.4% of the participating German ICUs treated COVID-19 patients in a separate ICU or in a separate area of an ICU. 22.6% of the ICUs can accommodate patients in single rooms with airlock. 10.9% used cohort isolation without an airlock (see Figure 1 (Fig. 1)).

### Prophylactic antibiotic administration

18.7% of responding ICUs regularly performed antibiotic prophylaxis in COVID-19 patients. 4.3% claimed to always use prophylactic antibiotics, while 42.4% did not indicate prophylactic antibiotics at all (see Figure 1 (Fig. 1)).

### Personal protective equipment

FFP2 were worn as standard masks in 83.2% of ICUs. 81.0% used additional isolation measures such as videolaryngoscopy and intubation drape frames [30]. At the time of the survey, a full-body suit was worn in only 9.5% of the units.

### PCR/isolation measures

PCR testing was used as the gold standard by 94.8% of ICUs to identify possible SARS-CoV-2 cases. Only 3.7% of ICUs exclusively tested symptomatic patients. PCR testing was also used to discontinue isolation measures in 97.8% of facilities. Radiologic testing or rapid antigen testing was used only to a small extent.

### Restriction of measures on patients

More than 90% of the patients received physiotherapy and mobilization measures. Visits by relatives took place in 19.7% of ICUs. However, when comfort care was initialized, relatives were allowed to visit in 65.7% of cases and in 84.7% during the immediate end-of-life care. Psychological support was available in 35.0% of ICUs.

## Discussion

### Isolation measures

Cohort isolation is common practice in many hospitals. Especially for well-studied infectious diseases, it seems to be safe for patients with the same pathogens to be isolated and cared for together, if architecturally necessary [31], [32]. This brings organizational advantages and cost savings, while mitigating negative psychological consequences of isolation for patients with infectious diseases. During the SARS-CoV-2 pandemic, many hospitals also implemented or prepared for cohort isolation for capacity reasons [33], [34]. Participants in our survey reported isolating patients with or without the usage of an airlock in 32.1% of cases. Thus, the vast majority of participating ICUs used some form of cohort isolation (for example, stand-alone isolation units, cohort isolation with or without an airlock or other measures). In light of the increasing prevalence of VoCs, cohort isolation should be discussed critically. For example, co-infection with another variant would be conceivable. Furthermore, bacterial superinfections occur, which may include drug-resistant germs [35], [36]. In these cases, cohort isolation is not sufficient; further infection control measures must be taken or individual isolation must be applied, and cohorting must be discontinued. Switching from one isolation mode to another also requires additional staff. The RKI recommends single rooms if there is risk of another pathogen or superinfection [32]. In addition, it should be noted that it is not always clear at the beginning which variants are involved, and that further variants are likely to occur as the pandemic progresses [37].

### Prophylactic antibiotic administration

The results of the present study suggest an unclear indication for prophylactic antibiotic administration in patients with SARS-CoV-2 infection. The current German level 3 guideline does not recommend prophylactic antibiotic therapy in diagnosed SARS-CoV-2 infection [5]. In their rationale, the guideline committee refers to a rapid review by Rawson et al. [38], in which bacterial coinfections are considered rare complications. However, it is clearly stated there that the supporting evidence is insufficient and should urgently be generated [38], [39]. This also seems evident when considering that about one-fifth of ICUs in our study regularly administer antibiotic prophylaxis to COVID-19 patients. Further studies are needed to investigate the impact on the overall prognosis and associated risks, as well as to implement a unified approach to prophylactic antibiotic administration based on specific indication criteria.

### Personal protective equipment

The benefit of PPE for healthcare workers in the management of COVID-19 patients has been proven beyond doubt [40], [41]. However, clinical implementation remains inconsistent. There are many reasons for this. For example, differences in standard operating procedures, different prerequisites, and also differences in the availability of PPE, at least during the beginning of the pandemic, come into play. In our study, 19% of participants reported wearing an FFP3 mask as the default face mask. The majority reported using FFP3 masks only in the context of high-risk activities (i.e., during aerosol-generating procedures). Similarly, the permanent wearing of protective eyewear or shields was established in only 73% of the participating ICUs. Only 81% of participating ICUs use advanced measures when manipulating the airways (these include videolaryngoscopy or the use of special devices to reduce aerosol formation). This is remarkable, as the recommendation to use videolaryngoscopy was made early in the course of the pandemic and should by now be considered a clinical standard in all participating hospitals [5].

### PCR/isolation measures

The determination of infection status with regard to SARS-CoV-2 was possible in most German hospitals early during the pandemic. However, some hospitals without an attached laboratory still rely on collaboration with external laboratories. For logistical reasons, timely access to a valid result is not always possible, and varied from 1 hour to 24 hours in our study (see Figure 2 (Fig. 2)). This implies a suboptimal utilization of isolation capacities, especially with regard to the limited numbers of ICU beds, since de-isolation may be delayed based on late laboratory results. One option to remedy this could be the use of on-site PCR devices that are able to produce rapid results. Although these are associated with higher costs, it might be cost-effective if measures such as de-isolation can be applied sooner [15]. Testing by PCR to lift isolation is currently the scientific consensus in severe cases, as is usually the case in intensive care units. In the case of VoCs, the RKI [15] also recommends additional testing for release from isolation. In this case, this should be carried out after 14 days. Hence, whether using on-site PCR testing is indeed cost-effective still remains unknown, particularly since quality management issues and validity of test results need to be considered as well. Accredited in-hospital laboratories are usually able to produce results faster and often significantly cheaper than on-site solutions. Furthermore, de-isolating of patients and the subsequent reoccupation of ICU beds usually requires considerable amounts of time. This could be further complicated by organizational aspects, such as changing shifts and staffing depending on the time of day (e.g., night or day shift). Hence, a faster test result does not always translate directly into faster de-isolation, since this is a multifaceted process.

### Restriction of measures on patients

Due to the strict isolation measures, parts of the usual supportive therapy, such as spiritual counseling, psychological support and visits by relatives were partly unavailable to the critically ill in a curative setting. However, we were able to show that especially in palliative situations, attempts were made to provide psychosocial and spiritual support to patients, e.g., by means of online communication. However, it should be reiterated that psychological care was available in a maximum of 35% of ICUs. This figure is alarmingly low, considering that the severe illness, stay in an ICU, and isolation measures represent a heavy psychological burden [42], [43]. There should be awareness of the importance of psychological care despite the scarcity of resources.

### Limitations

A frequent limitation of survey studies is the low response rate and the resulting lack of representativeness. In the present study, we assume that the response rate appears artificially low. This is due to the fact that although 1,340 ICUs are included in the DIVI registry, not all of them actually treat COVID-19 patients. It should be noted that although only some of the German ICUs were able to participate in the study, a clear trend is discernible. We would also like to point out that the questions in this study cover a wide range, since they were asked as part of a larger study. Many more questions arise and we do not claim to provide a complete account of the issues in the context of this study.

## Conclusions

We were able to identify differences in the use of PPE, antibiotic prophylaxis, and isolation measures. We showed that, despite difficult circumstances, German ICUs try to provide holistic treatment to their critically ill patients, especially during palliative care. Implementation of the recommendations for the treatment of patients with COVID-19 is inconsistent. We consider the survey a good opportunity to assess the extent to which the guidelines are being followed. In cases of high deviation, the findings should be examined in more detail. It is possible that the evidence in these cases is inconclusive. Further surveys should be conducted to track adherence to the guidelines and highlight areas that need further evaluation.

## Notes

### Competing interests

The authors declare that they have no competing interests.

### Acknowledgments

We would like to thank all the intensive care units that participated in our survey, making this study possible. 

### Authorship

All authors support the current recommendations of the International Committee of Medical Journal Editors (ICMJE). All authors read and approved the final manuscript.

Steffen Dickel and Clemens Grimm contributed equally to this work.

### Funding

This study was made possible within the framework of the CEOsys network. The CEOsys project is funded by the German government's Federal Ministry of Education and Research (BMBF); Funding number FKZ 01KX2021.

### Privacy/ethics statement

Participation in this study was voluntary. It is not possible to attribute the survey data to a single participating clinic. The survey was therefore completely anonymous. The entry of an email address was possible separately, without connection to the survey data. In retrospect, the address of the clinics could be collected via email to establish further research.

## Figures and Tables

**Table 1:**
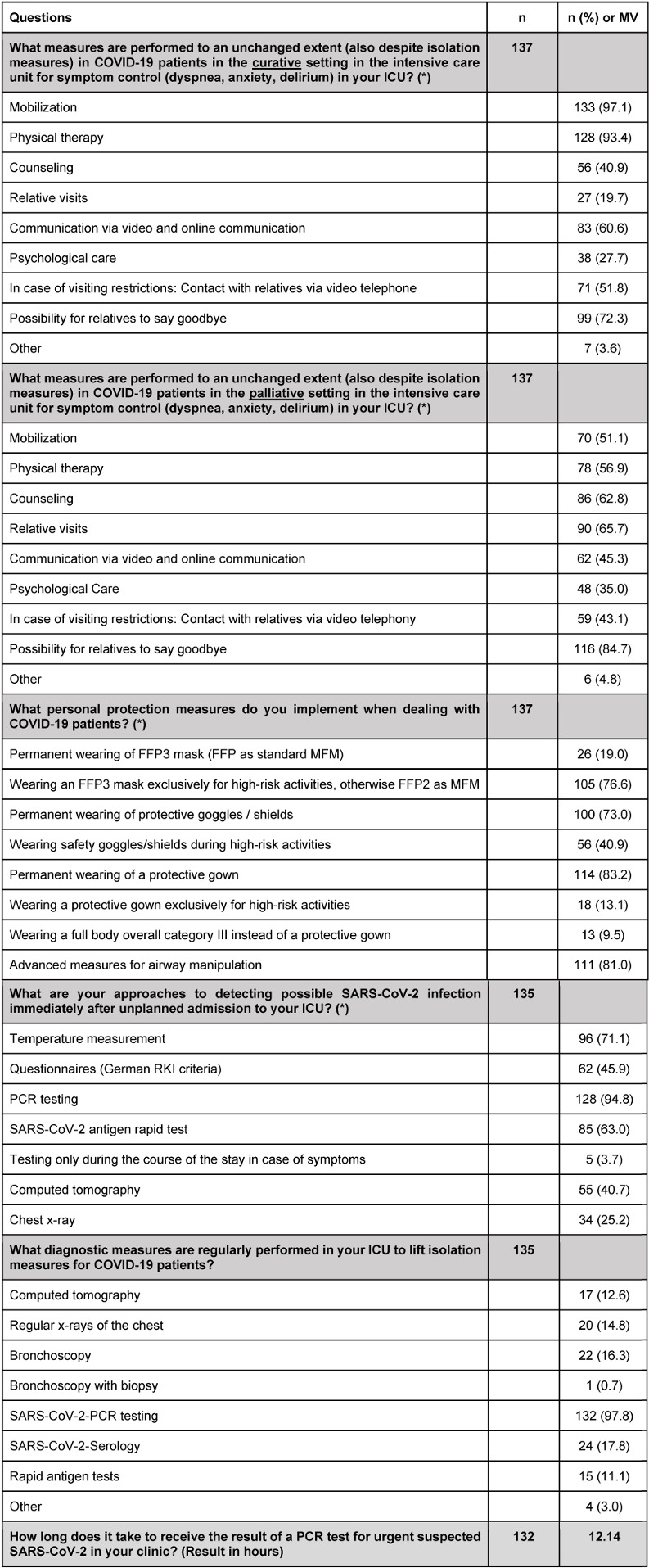
Results of the online survey with absolute numbers and in percent. The time taken to obtain the results of the PCR test is shown as the mean value (MV) in minutes.
